# Green Synthesis of Multifunctional Carbon Dots with Antibacterial Activities

**DOI:** 10.3390/nano11020369

**Published:** 2021-02-02

**Authors:** Arumugam Saravanan, Moorthy Maruthapandi, Poushali Das, John H. T. Luong, Aharon Gedanken

**Affiliations:** 1 Department of Chemistry, Bar-Ilan Institute for Nanotechnology and Advanced Materials (BINA), Bar-Ilan University, Ramat-Gan 52900, Israel; saran.bc94@gmail.com (A.S.); lewismartin.jesus@gmail.com (M.M.); das.poushali91@gmail.com (P.D.); 2 School of Chemistry, University College Cork, T12 YN60 Cork, Ireland; luongprof@gmail.com

**Keywords:** *Curcuma longa*, carbon dots, fluorescence, photostability, bactericidal property

## Abstract

Carbon dots (CDs) were obtained from medicinal turmeric leaves (*Curcuma longa*) by a facile one-step hydrothermal method and evaluated for their bactericidal activities against two gram-negative; *Escherichia coli*, *Klebsiella pneumoniae*, and two gram-positive counterparts; *Staphylococcus aureus*, *S. epidermidis*. The CDs exhibited spherical shapes with a mean size of 2.6 nm. The fluorescence spectra of CDs revealed intense fluorescence at λ_ex_/_em_ = 362/429 nm with a bright blue color in an aqueous solution. The CDs showed strong photostability under various environmental conditions (pH, salt, and UV-radiation). The complete bactericidal potency of CDs was 0.25 mg/mL for *E.coli* and *S. aureus* after 8 h of exposure, while for *K. pneumoniae*, and *S. epidermidis*, the CDs at 0.5 mg/mL good antibacterial effect within 8 h and complete eradication after 24 h of exposure is observed. The release of reactive oxygen species played a crucial role in the death of the bacterial cell. The present study provides a strategy for the preparation of CDs from a medicinal plant and their potential antibacterial activities against four common contagious pathogens.

## 1. Introduction

Luminescent carbon nanodots (CDs, C-dots) were accidentally discovered during the refining of single-walled carbon nanotubes in 2004 [1]. Subsequently, the preparation of fluorescent carbon nanoparticles called CDs with a dimension of <10 nm was materialized [2]. CDs, a new-fangled member of the carbon nanomaterials, have good chemical and photochemical stability together with biocompatibility without intrinsic toxicity. Their unprecedented and unique properties encompass tunable emission, optical properties, biocompatibility, nontoxic, superior quantum yield, water solubility, and up-conversion photoluminescent, etc. [3,4,5,6]. There have been several efforts to apply fluorescent CDs into the field of sensors [7], optoelectronics [3], energy storage [8], light-emitting diode (LED) [9], drug delivery [10], antibacterial [11,12], bioimaging [13,14], catalysis [15], etc. To date, different synthetic routes have been identified to prepare CDs including hydrothermal [16,17], solvothermal [18,19], ultrasonication [20,21,22], simple heating [23,24], arc discharge [25], microwave-assisted pyrolysis [26,27], laser ablation [2,28], electrochemical [29,30], etc. Initially, carbonaceous materials were utilized for the preparation of CDs resulting in lower quantum yields (QY) and limited solubilities. Considerable efforts have been taken to overcome these two drawbacks, the development of green synthetic methods for CDs has received considerable consideration. To acquire CDs through a facile, low-cost, eco-friendly method with unique properties, different natural carbon precursors have been studied such as potato [31], orange juice [32], grass [33], oats [34], *Allium sativum* peel [35], etc.

In recent days, the synthesis of CDs from medicinal plants have been attracted due to their cost effectiveness, availability, and natural phytocompounds. In this regard, leaves from medicinal plants such as tulsi [36], coriander leaves [37], aloe vera [38], etc. have been utilized for the synthesis of CDs synthesis due to their numerous phytochemicals with greater therapeutic values. CDs from medicinal plants are free from toxic chemicals and have natural capping agents. Turmeric (*Curcuma longa* L.) is a major herb mostly cultivated in Asia and widely used as a spice and coloring agent or food additive. It has become a popular medicinal plant worldwide, curcumin is one of the major compounds in turmeric and it functions as a drug with antioxidant, anti-bacterial, anti-fungal, anti-parasitic, anti-inflammatory, anti-mutagenic, anti-carcinogenic, and detox properties [39].

Inspiring by the above biological properties, herein we extend their antibacterial properties by making CD nanoparticles (without a passivating agent) from turmeric (*Curcuma longa*) leaves by a one-step green synthetic pathway. The major phytochemical in turmeric leaves like curcumin, demethoxycurcumin, and bisdemethoxycurcumin serve as the carbon sources. Turmeric leaves are easily available, cheap, and can be readily converted to CDs by a simple hydrothermal technique. This work reveals a green, reliable method for the preparation of cost-effective photoluminescent CDs. Their potential applications as an emerging antimicrobial agent against four common bacterial strains are reported in the current manuscript.

## 2. Experimental Section

### 2.1. Materials

Turmeric leaves (*Curcuma longa*) were obtained at the green stage in the local area, Israel. Ultrapure water from Alfa Aesar (Haverhill, MA, USA) was used for the preparation of CDs. *E. coli* (ATCC 25922), *S. aureus* (ATCC 29213), *K. pneumoniae* (ATCC 700603), and *S. epidermidis* (ATCC 12228) were obtained from Dr. Banin’s Lab, The Mina and Everard Goodman Faculty of Life Science, Bar Ilan University, Israel.

### 2.2. Preparation of CDs

CDs were successfully achieved by the one-step hydrothermal method. Briefly, 5 g of turmeric fresh leaves were washed, ground with 40 mL of ultrapure water in a mortar pestle. The leaves paste was then extracted with 20 mL of water. The solution was heated in a hydrothermal cell at 180 °C for 10 h and then continued cooling down to ambient temperature. The deep brown color extract was centrifuged at 8000 rpm for 20 min. The final yellow color solution was filtered and dialyzed with a cellulose syringe filter and a dialysis bag (MW cut-off = 500–1000). The final product was powdered using lyophilizer and dissolved in water.

### 2.3. Characterization of CDs

Transmission electron microscopy (TEM) image was collected with TEM- JEOL-2100 (Peabody, MA, USA), and the sample for TEM was prepared by dropping the aqueous solution of CDs onto a copper-coated TEM grid and dried at 50 °C for 2 h. UV-Visible (Varian Cary 100 Bio Spectrophotometer) and fluorescence spectrophotometers (Varian Cary Eclipse) were used for optical properties measurement. Fourier transform infrared (FTIR) spectra were recorded using a Tensor 27 spectrometer (Bruker, Germany) ranging from 500 to 4000 cm^−1^. The surface charge was examined with Malvern Zetasizer Nano-ZS (Malvern, UK). X-ray photoelectron spectroscopy was carried out on an XPS, Nexsa spectrometer (England). The binding energies were tuned by the C1s peak at 285eV for all elements. The ROS generation of CDs was detected on a Bruker X-band spectrometer (121 EPR 100d) using DMPO (5,5-dimethyl-1-pyrroline-N-oxide) as a spin trap. A 40 μL of the CD solution was admixed with 10 μL of DMPO (0.01 M) for EPR measurement. The blank was measured using DI water without CDs.

### 2.4. Photostability of CDs

The pH stability was analyzed by mixing 2 mL of buffer at different pHs with 1 mL of CDs (1 mg/mL). For ionic strength, 2 mL of a solution with various concentrations of NaCl (0–1.0 M) were added with 1 mL of CDs (1 mg/mL). The FL emission of the solutions was recorded at an emission wavelength of 429 nm after 10 min.

### 2.5. Antibacterial Activity Test

*E. coli* (gram-negative), *K. pneumoniae* (gram-negative), *S. aureus* (gram-positive), and *S. epidermidis* (gram-positive) were grown in lysogeny broth (LB) at 37 °C overnight with agitation at 180 rpm. The resulting bacterial concentration in LB broth was adjusted to 10^7^ by taking absorbance at 595 nm (OD^595^). Typically, 500 μL of bacterial cells (10^7^) in LB was mixed with 500 μL of CDs with varying concentrations (0.25, 0.5, and 1.0 mg/mL) and incubated at 37 °C for 8–24 h with shaking at 120 rpm. The appropriate amount of incubated bacterial solution was consecutively diluted and plated on an agar coated plate. The colony-forming unit (CFU) method was followed to calculate the rate of bacterial growth.

## 3. Results and Discussion

### 3.1. Surface Morphology and Optical Properties

**Figure 1:**
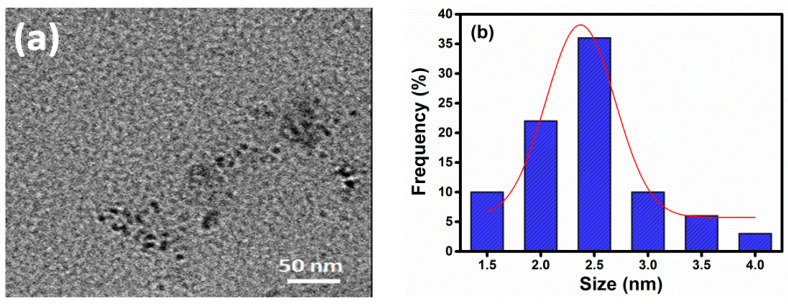

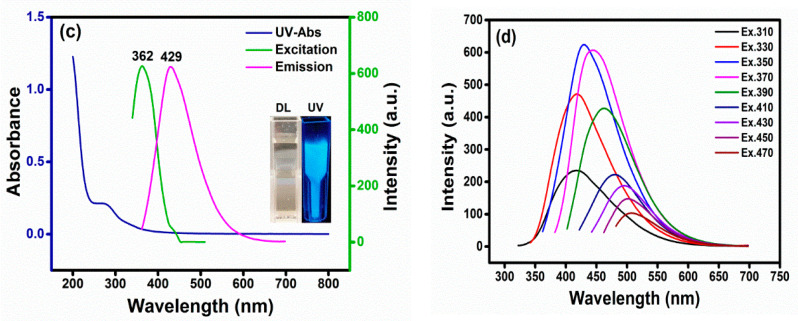
(**a**) TEM images, (**b**) FT-IR spectra, (**c**) UV, Maximum λ_ex_ and λ_em_ (Inset: photo of CDs under normal light and UV-light), and (**d**) fluorescence spectra of CDs at diverse excitation.

The TEM image shows that CDs appeared as a spherical shape with an average mean particle size of 2.6 nm (Figure 1a,b). Subsequently, the particle cluster was ranging from 1.5 to 4.0 nm (Figure 1b) as confirmed by the particle analysis tool of image J software. The UV absorption spectra (Figure 1c) show a characteristic peak at 288 nm, because of π-π* interaction of C=C and C–C bonds, and there is no n- π* transition between 300–400 nm. The synthesized CDs exhibited the strongest emission at 429 nm with excitation at 362 nm (Figure 1c). The CDs in aqueous solution appeared as a light yellow color under daylight (Figure 1c, inset), but exhibited bright blue luminescence when irradiated with UV light at 350 nm. The fluorescence intensity of CDs has a maximum at 429 nm when photoexcited at 350 nm, showing the typical fluorescence nature of CDs. The different emission spectra of CDs were obtained by increasing the range of excitation value from 310 to 470 nm with a 20 nm interval (Figure 1d). The excitation at 350 nm was preeminent to produce a bright color fluorescence at 429 nm. Like CDs from a natural source, these CDs also exhibited excitation dependent FL behavior (Figure 1d), owning to the abundance of different sized nanoparticles and functional groups on the surface of the CDs as well as defects of CDs [40].

### 3.2. FTIR and XPS

**Figure 2:**
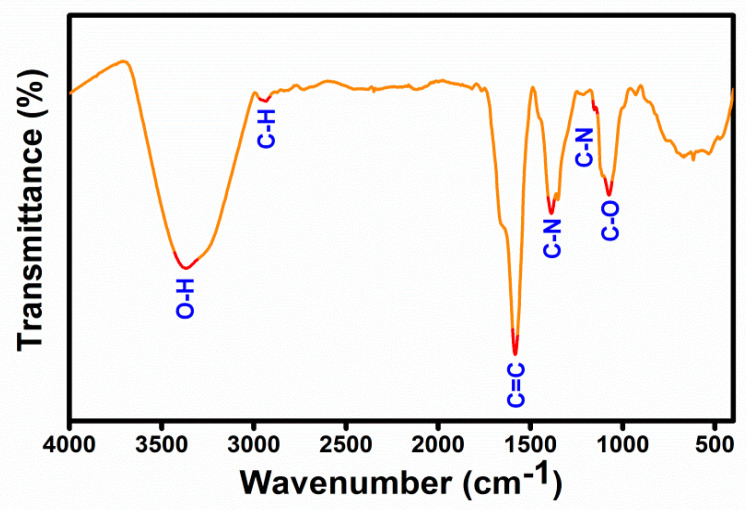
FTIR spectra of as-prepared CDs.

FTIR spectra were employed to explore functional groups in CDs (Figure 2). The FT-IR spectra showed a strong band at 3370 is caused by O-H stretching of hydroxyl groups of phenol, this band is observed also for aliphatic OH whereas a moderate peak at 2951 cm^−1^ is assigned to C–H stretching vibration [41]. A strong peak at 1583 cm^−1^ reflects the -C=C- stretching of phenolic/aromatic rings. The two peaks at 1386 cm^−1^ and 1153 cm^−1^ represent the stretching vibration of C–N [36]. The bending and stretching vibration of C–O bonds in the hydroxyl group is denoted at 1073 cm^−1^.

**Figure 3:**
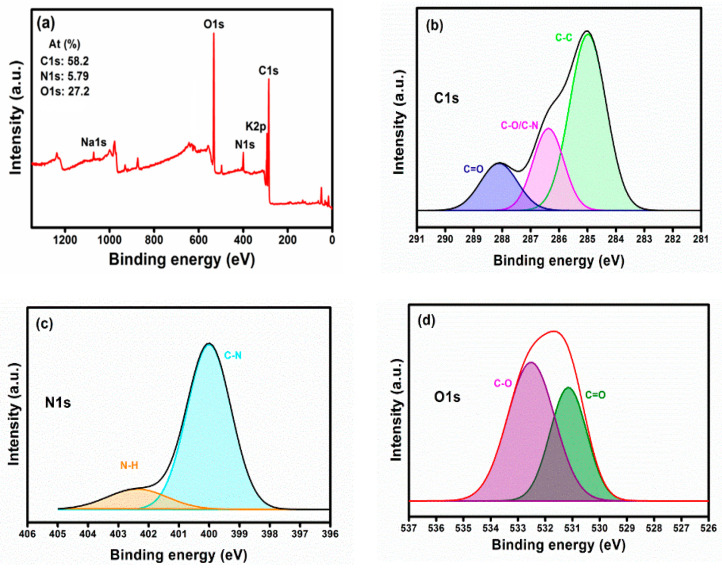
XPS full spectra (**a**), high-resolution spectra of C1s (**b**), N1s (**c**), and O1s (**d**) of CDs.

The major elemental composition of CDs was C = 58.2, N = 5.79, and O = 27.2% as determined by XPS analysis. The full XPS spectra of CDs (Figure 3a) show C1s, N1s, and O1s at 285, 400, and 532 eV, respectively. Other smaller peaks at 293 and 1069 eV are accredited to metal elements K2p and Na1s, respectively. These trace levels of mineral elements are anticipated to stem from the plant leaves. The deconvoluted high-resolution spectra of C1s (Figure 3b) presented three distinct peaks located at 284.9, 286.3, and 288.1 eV, corresponding to C–C, C–O/C–N, and C=O functional groups, respectively [42,43,44]. The two peaks at 400.1 and 402.3 eV in high-resolution spectra of N1s, which can be attributed to the presence of C–N and N-H groups, respectively [45]. In the O1s high-resolution spectra, two peaks at 531.2 and 532.5 eV are assigned to C=O (carbonyl) and C–O bonding [46]. The FTIR and XPS results suggest that the CDs have been synthesized through this facile and green hydrothermal treatment without the use of any additional chemicals/dopants. The purified CDs are hydrophilic since they have various functional groups, including carbonyl (C=O), hydroxy (–OH), carboxylic acid (O–C=O), and secondary amine (N-H) groups. These functional groups could be derived from phenolic and aromatic compounds (curcumin, demethoxycurcumin, and bisdemethoxycurcumin) in turmeric leaves [47].

### 3.3. The Photostability of Fluorescent CDs

**Figure 4:**
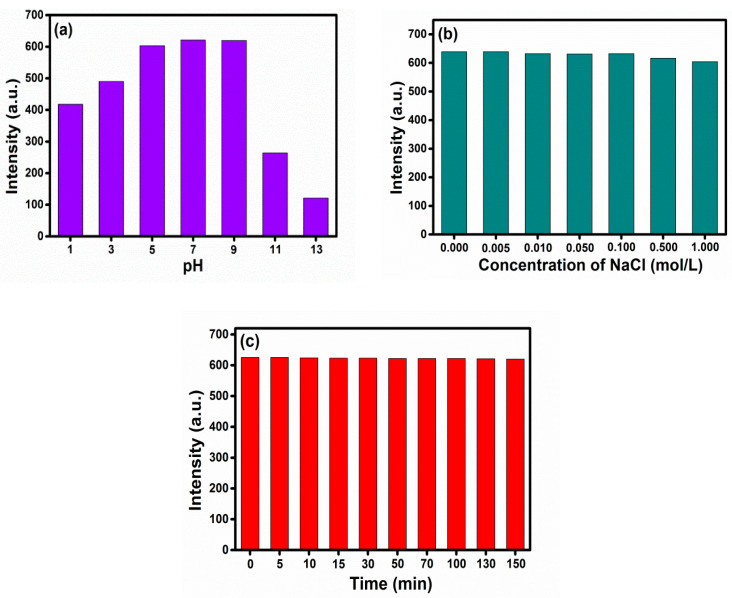
Photostability of CDs under (**a**) pH (**b**) NaCl, and (**c**) UV-light.

The photostability of CDs under pH and salt conditions is a significant property that can alter the optical and structural behavior of CDs. In this work, pH, ionic strength, and UV-radiation on the fluorescence intensity of CDs were conducted. As depicted in Figure 4a, the emission intensity was increased significantly following the decrease in the acidic condition while a decreasing trend was observed at the extreme alkaline condition. The fluorescence emission of CDs was reduced by 57–80% in the pH = 11 to 13 due to the deprotonation of CDs. The emission intensity at pH-7–9 was optimal, considering its importance for practical bioapplications. A negligible (5%) decline in FL emission of CDs was observed at a high concentration of NaCl (0.5 M), compared to their behavior in deionized water (Figure 4b). Further, CDs were photostable under continuous UV radiation (a xenon arc lamp) at λ_ex/em_ = 340/429 nm for 150 min (Figure 4c).

### 3.4. Antibacterial Activity

**Figure 5:**
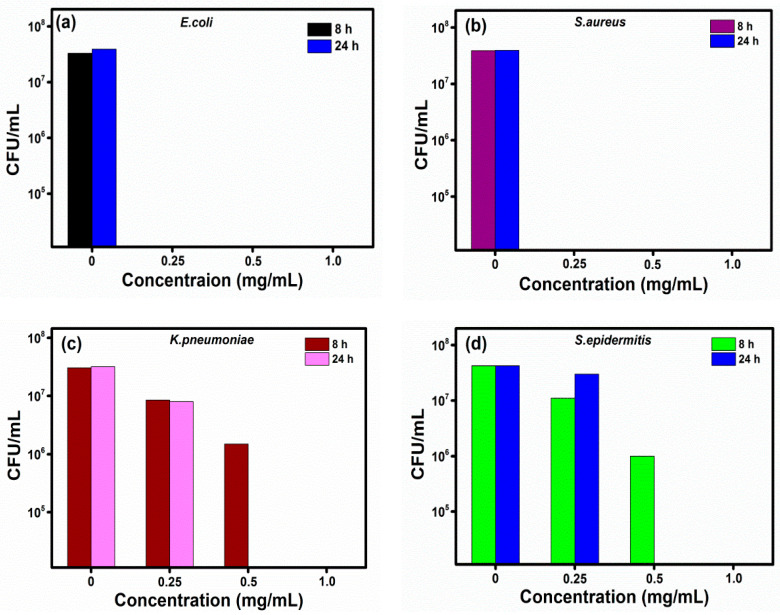
Bactericidal activity of CDs on (**a**) *E. coli*, (**b**) *S. aureus*, (**c**) *K. pneumoniae,* and (**d**) *S. epidermitis.*

The synthesized CDs was studied their antibacterial activity against four various gram-positive and gram-negative bacteria. The CDs were demonstrated superior for the eradication of gram-positive bacteria of *S*. *aureus* and *S. epidermidis* and gram-negative *bacteria E. coli* and *K. pneumoniae.* The minimum inhibitory concentration (MIC) is 0.25 mg/mL for *E. coli* and *S. aureus* and 0.5 mg/mL is for *K. pneumoniae* and *S. epidermidis*. The effectivity of CDs on the growth inhibition of *E. coli* and *S. aureus* within 8 h at 0.25 mg/mL (Figure 5a,b), compared to 0.5 mg/mL for *K. pneumoniae* and *S. epidermidis*. However, the complete eradication of bacterial cells was observed after 24 h incubation with 1 mg/mL of the CDs (Figure 5c,d). Both pathogens *E. coli* and *S. aureus* were completely eradicated even at low concentration (0.25 mg/mL) with 8 and 24 h incubation, respectively, however, the pathogens *K. pneumoniae* and *S. epidermidis* required maximum incubation time and CDs concentration (1 mg/mL for 24 h) to attain complete growth inhibition. The results indicated that the CDs have a rapid response to growth inhibition on *E. coli* and *S. aureus* with low concentration and incubation period. Nevertheless, *K. pneumoniae* and *S. epidermidis* were displaying a much higher incubation time for the complete eradication. In comparison to other heteroatom doped CDs reported in the literature [48,49,50], our synthesized CDs exhibited superior antimicrobial effects against all four pathogens without any passivation of atoms. Two major compounds, demethoxycurcumin and bisdemethoxycurcumin, are partially remained inside or on the surface of CDs, which enhance the bactericidal property of CDs. The cytotoxicity of CDs in water was evaluated by the standard MTT colorimetric assay. The cell viability assay was also performed on the PC-3 cell line to detect the inherent cytotoxicity of CDs. Figure S1 (Supplementary Materials) shows that cell survival remained greater than 95% even at 200 μg/mL of CDs after incubation for 24 h. However, CDs at 500 μg/mL reduced 50% of cell viability after 24 h incubation. The antibacterial activity of this study was comparatively displayed with previous literature in Table 1. The antibacterial effectivity of this study was comparatively displayed with the previous literature in Table 1.

### 3.5. A postulated Mechanism

**Figure 6:**
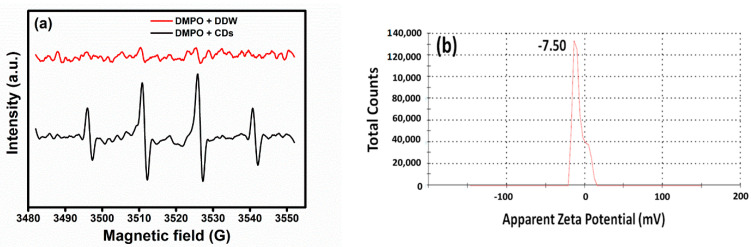
(**a**) ESR spectra and (**b**) surface charge of CDs.

A possible mechanism behind the observed cell death is related to reactive oxygen species (ROS). It was investigated by measuring electron paramagnetic resonance (EPR) for the samples. The EPR measures the ROS by DMPO (spin trap). The spin trap detects superoxide and hydroxyl radicals of HO-DMPO resulting in a distinct quartet peak with 1:3:3:1 signal intensity for OH radicals (Figure 6a). There was a 2-fold and 3.5-fold increase in the reactive oxygen species of the CDs, compared to the control. Thus, the ROS production by CDs played an important role in the eradication of the four tested bacteria. The CDs with unpaired or free electrons can react with the dissolved oxygen in the solution to produce additional ROS [51,52].

The synthesized CDs have a negative charge on their surface due to the presence of carboxylic acid and carbonyls on the surface as confirmed by XPS spectra (Figure 3). Pristine CDs have a zeta potential of −7 mV (Figure 6b); thus, electrostatic interactions are unlikely a major role in the eradication of the tested bacteria. Both gram-negative and gram-positive bacteria have membranes with negative charges, however, their interaction with slightly negatively charged surfaces is still possible due to other van der Walls forces, consisting of weak London dispersion forces and stronger dipole-dipole forces [52].

## 4. Conclusions

In summary, we have synthesized multifunctional CDs from natural source turmeric (*Curcuma longa*) leaves without any chemicals by a facile one-step hydrothermal process. The turmeric leaves are easily available, making the production of the CDs cost-effective. They have various secondary metabolites and served as a carbon source for preparing CDs. The CDs exhibited superior photostability under various environmental conditions. CDs can eradicate four different types of gram-negative and gram-positive bacteria. The bactericidal property of CDs on both *E. coli* and *S. aureus* was at low concentration and incubation time (0.25 mg/mL for 8 h). However, *K. pneumoniae* and *S. epidermitis* required 1 mg/mL and 24 h incubation to achieve complete growth inhibition. Our finding may open a gateway to synthesize naturally derived CDs from turmeric leaves towards a new antimicrobial agent.

## Figures and Tables

**Table 1:** Comparison table on antibacterial activity of CDs of the present study with previously reported literature.

Type of CDs	Source	Method	Bacteria Tested	MIC/ZOI Value	Exposure Time (h)	Ref.
OCDs TCDs	Tea leaves and milk vetch	Hydrothermal	*E. coli and S. aureus*	1000 μg/mL	4	[45]
ACDs	*A. argyi* leaves	Ignited	*E. coli*	150 μg/mL	24	[46]
CDs	chlorhexidine gluconate	Hydrothermal	*S. aureus* *E.coli*	150 μg/mL	24	[49]
Ag@CDs	*C. sativa* leaf AgNO_3_	Stirring	*E. coli and S. aureus*	45 μg/mL	-	[50]
CDs	*L. inermis* leaves	Hydrothermal	*E. coli* *S. aures*	5000 μg/mL	24	[53]
CDs	Oyster mushroom	Hydrothermal	*S. aureus, * *K. pneumoniae*	30 μg/mL	24	[54]
NCQDs	D-Glucose, Diethylenetriamine	Hydrothermal	*S. epidermidis*	0.5 mg/mL	18	[55]
CDs	Turmeric leaves leaves	Hydrothermal	*E. coli*	0.25 mg/mL	8	This work
			*S. aureus,*	0.25 mg/mL	8	
			*K. pneumoniae*	1 mg/mL	24	
			*S. epidermidis*	1 mg/mL	24	

## Data Availability

Data presented in this study are available by requesting from the corresponding author.

## Supplementary Materials

The following are available online at https://www.mdpi.com/2079-4991/11/2/369/s1, Figure S1: Cell viability of PC-3 cells after 24 h incubation with various concentrations of CDs.
