# Supplementary material for: Green Synthesis of Multifunctional Carbon Dots with Antibacterial Activities
Source: Nanomaterials (Basel). 2021 Feb 2;11(2):369. doi: 10.3390/nano11020369 (PMC7912860; doi:10.3390/nano11020369)
Supplement: Supplementary file 1 [file nanomaterials-11-00369-s001.pdf]

Supporting Information

Green Synthesis of Multifunctional Carbon Dots with Antibacterial Activities

Arumugam Saravanan [1], Moorthy Maruthapandi [1], Poushali Das [1], John H.T. Luong [2] and Aharon Gedanken [1,*]

[1]Department of Chemistry, Bar-Ilan Institute for Nanotechnology and Advanced Materials (BINA), Bar-Ilan University, Ramat-Gan 52900, Israel; saran.bc94@gmail.com (A.S.); lewismartin.jesus@gmail.com (M.M.); das.poushali91@gmail.com (P.D.)
[2]School of Chemistry, University College Cork, T12 YN60 Cork, Ireland; luongprof@gmail.com
* Correspondence: gedanken@mail.biu.ac.il

Cell viability assay

Cell viability assay was determined by the MTT colorimetric method using the tetrazolium dye MTT 3-(4,5-dimethylthiazol-2-yl)-2,5-diphenyltetrazolium bromide. PC-3 cells were cultured at 37 °C in a 5 % CO_2 in L-glutamine RPMI 1640 medium with 10% fetal bovine serum (FBS) and 1% penicillin-streptomycin-nystatin (Biological Industries, Beit Haemek, Israel). Cell viability was determined using an MTT colorimetric assay on PC-3 (8000 cells/well) cell line. The PC-3 cells were exposed with CDs with diverse concentrations (100–500 μg/mL) for 24 h incubation. The MTT reagent was added and incubated for 2-4 h. The color intensity was measured at 570 nm by an ELISA plate reader. The cell viability in percentage was calculated as % Cell viability = [A_{570nm} of treated cells/ A_{570nm} of control cells] X 100.

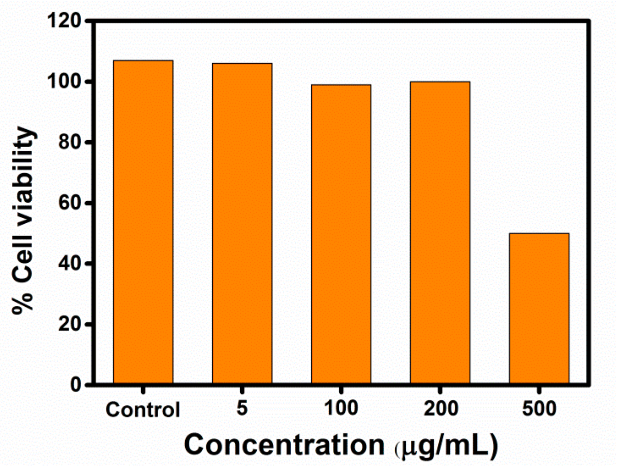

Figure S1. Cell viability of PC-3 cells after 24 h incubation with various concentrations of CDs
